# Normal values for native T_1_ at 1.5 T in the pericardial fluid of healthy volunteers

**DOI:** 10.1093/ehjimp/qyad028

**Published:** 2023-09-19

**Authors:** Simon Thalén, Joao G Ramos, Henrik Engblom, Andreas Sigfridsson, Peder Sörensson, Martin Ugander

**Affiliations:** Department of Clinical Physiology, Karolinska Institute, Karolinska University Hospital Solna, 171 76 Stockholm, Sweden; Department of Clinical Physiology, Karolinska Institute, Karolinska University Hospital Solna, 171 76 Stockholm, Sweden; Department of Clinical Physiology, Karolinska Institute, Karolinska University Hospital Solna, 171 76 Stockholm, Sweden; Department of Clinical Physiology, Karolinska Institute, Karolinska University Hospital Solna, 171 76 Stockholm, Sweden; Department of Cardiology, Karolinska University Hospital, Stockholm, Sweden; Department of Clinical Physiology, Karolinska Institute, Karolinska University Hospital Solna, 171 76 Stockholm, Sweden; Kolling Institute, Royal North Shore Hospital, and Charles Perkins Centre, University of Sydney, St Leonards, Sydney, NSW 2065, Australia

**Keywords:** pericardial fluid, native T_1_, T_1_ mapping, healthy volunteers

## Abstract

**Aims:**

T_1_ mapping cardiovascular magnetic resonance (CMR) imaging has been used to characterize pericardial effusions. The aim of this study was to measure pericardial fluid native T_1_ values in healthy volunteers to establish normal values.

**Methods and results:**

Prospectively recruited volunteers (*n* = 30) underwent CMR at 1.5 T, and native T_1_ maps were acquired using a modified look-locker inversion recovery 5s(3s)3s acquisition scheme. A volume of pericardial fluid was imaged in a short-axis slice and in a slice perpendicular to the short-axis orientation. A reliable measurement had a region of interest (ROI) size > 10 mm^2^, coefficient of variation < 10%, and a relative difference < 5% between the two slice orientations. In 26/30 (87%) of volunteers, there was a sufficient amount of pericardial fluid to enable reliable measurement. Native T_1_ of pericardial fluid was 3262 ± 163 (95% normal limits 2943–3581 ms) and did not differ in the perpendicular slice orientation (3267 ± 173 ms, *P* = 0.75), due to sex (female 3311 ± 177 vs. male 3220 ± 142 ms, *P* = 0.17), age (*R*^2^ = 0.03, *P* = 0.44), heart rate (*R*^2^ = 0.005, *P* = 0.7), or size of the ROI (0.06, *P* = 0.23).

**Conclusion:**

This study shows that T_1_ values can be reliably measured in the pericardial fluid of healthy volunteers. It is the first to report normal reference ranges for T_1_ values at 1.5 T in the pericardial fluid of healthy volunteers.

## Graphical Abstract

**Graphical Abstract:**
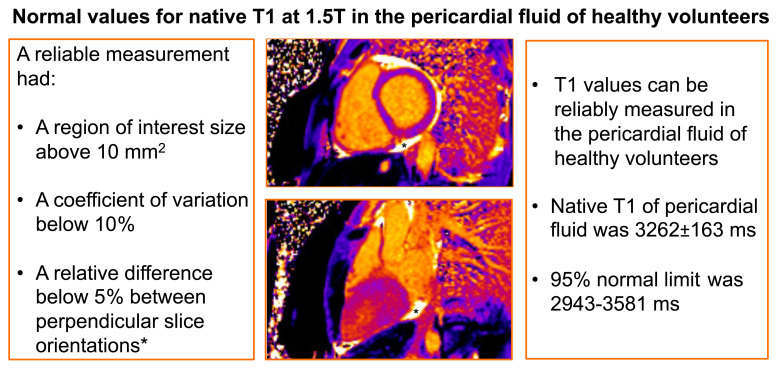

## Introduction

Pericardial fluid is a serous ultrafiltrate of plasma normally present between the layers of the pericardium enveloping the heart. An increase in the amount of fluid above ∼30–35 mL is considered a pericardial effusion.^1^ Pericardial effusions can have a wide variety of aetiologies and are a common clinical occurrence. The diagnostic approach includes categorizing the effusion as transudative, due to an imbalance in osmotic and hydrostatic pressures, or exudative, due to an increase in permeability of the pericardium secondary to local inflammation. Biochemical analysis may be performed and interpreted using the Light criteria, originally developed and validated for assessing pleural effusions.^2^

With the rapid development of new techniques in cardiovascular magnetic resonance (CMR) imaging, there have been several attempts to non-invasively characterize effusion fluids. Early attempts using magnetic field strengths below 1 T were largely unremarkable.^3–7^ Later results using diffusion-weighted imaging have shown some promise but also conflicting results.^8–11^

T_1_ mapping, a technique whereby the T_1_ value is quantified by imaging on a pixel-by-pixel basis, has now largely superseded the previous T_1_-weighted methods where the T_1_ value was encoded into arbitrary signal intensities and only indirectly estimated. T_1_ mapping CMR has enabled the characterization of focal and global cardiovascular pathologies and made it possible to reliably and quantitatively perform longitudinal and group comparisons.^12^

Non-contrast (native) T_1_ mapping has successfully been used to classify pericardial and pleural effusions as transudate or exudate^13^ and has further been used to successfully differentiate between malignant and non-malignant pleural and ascitic effusions *ex vivo.*^14^ The rationale for these approaches is that proteins and cells leaking through a permeable inflamed pericardium and into the pericardial fluid have paramagnetic properties that lower the T_1_ value of the effusion fluid. Consequently, a low native T_1_ value would be consistent with an exudate and a high value with a transudate.

The current study aimed to measure T_1_ in pericardial fluid in healthy volunteers by determining the feasibility of these measurements and establishing normal values.

## Methods

### Volunteers

Healthy volunteers (*n* = 30) with an absence of history of cardiovascular disease were recruited by local advertisement. Clinical assessment of CMR images was used to confirm the absence of myocardial or pericardial disease. Exclusion criterion was metallic implants not safe for magnetic resonance imaging.

### Image acquisition

T_1_ maps were acquired at end diastole using a modified look-locker inversion recovery (MOLLI) sequence^15^ at 1.5 T (MAGNETOM Aera, Siemens Healthcare, Erlangen, Germany) using motion correction.^16^ A 5s(3s)3s acquisition scheme was used where two inversions were made and images acquired once per heartbeat during 5 s after the first inversion, followed by a 3-s pause, and images acquired once per heartbeat during 3 s after the second inversion.^17^ Typical image acquisition parameters were as follows: flip angle 35°, matrix size 256 × 136–158, slice thickness 6 mm, initial inversion time 129 ms, field of view 300–410 × 241–384 mm^2^, and parallel imaging factor 2 for a voxel dimension of ∼1.5 × 1.5 × 6 mm.

**Figure 1:**
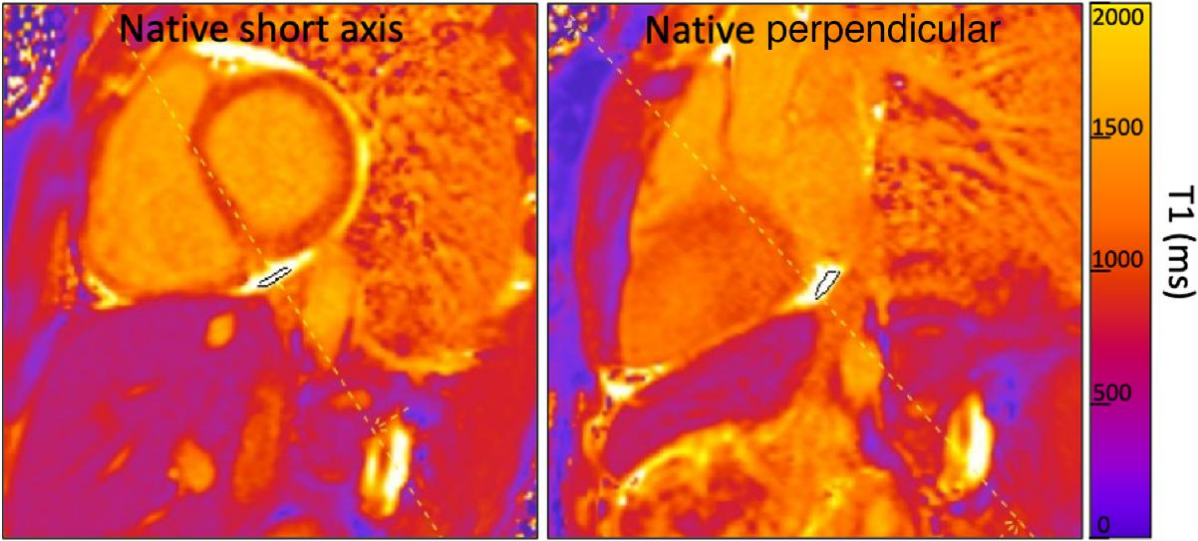
An example of native T_1_ maps taken in SA (left) and P (right) slice orientations.

A short-axis (SA) stack of T_1_ maps encompassing the heart and the ascending aorta was acquired and screened for the presence of pericardial fluid visible as areas with native T_1_ values above 2500 ms. A slice perpendicular (P) to the SA slice was then prescribed to enable two perpendicular measurements. An example of T_1_ maps from one volunteer is shown in *Figure 1*.

### Image analysis

Image analysis was performed on clinical workstations using clinical imaging software (syngo.via VB30A; Siemens Healthcare GmbH, Erlangen, Germany). Regions of interest (ROI) were manually prescribed in the same volume of pericardial fluid of both the SA and P slices. A 3D cursor feature was used to ensure measurement in the same volume of pericardial fluid in both slice orientations.

The anatomical location of the pericardial fluid, standard deviation (SD) of T_1_ values within the ROI, and its size were recorded. To evaluate measurement reliability, the following criteria were used to define a reliable measurement: (i) the ROI size needed to be >10 mm^2^ and <50 mm^2^, (ii) the coefficient of variation of measured values within the ROI needed to be <10%, and (iii) the relative difference in measured values between the SA and P slice orientations needed to be below 5%. The coefficient of variation was defined as the ratio of the SD to the mean. The relative difference in native T_1_ between SA and P measurements was defined as (SA − P)/[(SA + P)/2].

### Statistical analysis

The normality of distributions was assessed both visually using Q–Q plots and the Shapiro–Wilk test, and data were reported as median (interquartile range) or mean ± SD as appropriate. Mean value comparisons were made using parametric paired or unpaired *t*-tests as appropriate. Correlations were calculated using Pearson’s product moment correlation or Spearman’s rho correlation as appropriate. A *P*-value of less than 0.05 was considered statistically significant. Normal reference ranges were calculated as mean ± 1.96 × SD. Statistical calculations were performed using the software package R (R Core Team 2020, Vienna, Austria).

## Results

The healthy volunteers averaged 35 (26–44) years of age, 14 (46%) were female, and a reliable measurement was possible in 26/30 (87%) volunteers. Of the healthy volunteers, four (13%) did not fulfil the reliability criteria detailed above. Baseline variable for healthy volunteers are summarized in *Table 1*.

**Table 1:** Baseline variables for healthy volunteers

Characteristic	
Reliably measurable T_1_, *n*/*n* (%)	26/30 (87)
Heart rate	68 ± 9
ROI size	0.18 (0.14–0.27)
Female sex, *n*/*n* (%)	12/26 (46)
Age, years	35 (46–65)
Height, cm	174 ± 10
Weight, kg	71 ± 12
Body mass index, kg/m^2^	22 ± 2
Body surface area, m^2^	1.8 ± 0.2

Data are presented as numbers (*n*) and per cent, mean ± SD, or median (interquartile range).

**Figure 2:**
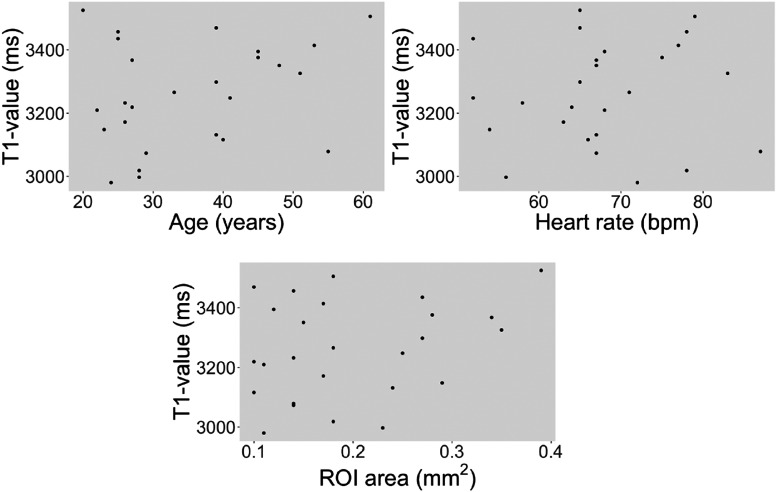
Scatterplots of T_1_ values from pericardial fluid and subject age, heart rate, and ROI size, respectively. There were no significant correlations, and hence, a line of regression has been intentionally omitted.

Native T_1_ of pericardial fluid was 3262 ± 163 (95% normal limits 2943–3581 ms) and did not differ in the P slice orientation (3267 ± 173 ms, *P* = 0.75). Native T_1_ of pericardial fluid did not differ due to sex (female 3311 ± 177 vs. male 3220 ± 142 ms, *P* = 0.17), age (*R*^2^ = 0.03, *P* = 0.44), heart rate (*R*^2^ = 0.005, *P* = 0.7), or size of the ROI (0.06, *P* = 0.23) as shown in *Figure 2*.

The localizations of reliably measurable pericardial fluid were the transverse pericardial sinus (*n* = 17, 65%), the inferior intraventricular sulcus (*n* = 5, 20%), and the oblique pericardial sinus (*n* = 4, 15%), and the limited number of observations per localization precluded statistical comparisons. No volunteer had reliably measurable T_1_ values in more than one localization.

## Discussion

This study shows that T_1_ values can be reliably measured in the pericardial fluid of healthy volunteers. It is the first to report normal reference ranges for T_1_ values in the pericardial fluid of healthy volunteers. Normal values are of importance as the pericardial fluid of healthy volunteers is usually not available for biochemical analysis.

The native T_1_ values of the pericardial fluid in the healthy volunteers in the present study are comparable to native T_1_ values in pericardial effusions presumed to be transudate.^13^ When applying the native T_1_ cut-off value proposed for exudates in that study (3105 ms), two healthy volunteers from the present study had a lower value.

The sequence used in this study was the 5s(3s)3s variant of MOLLI. Although other cardiac T_1_ mapping sequences are available such as SASHA,^18^ shMOLLI,^19^ and SAPPHIRE,^20^ MOLLI was chosen as it is the cardiac T_1_ mapping sequence most widely used clinically. The 5(3)3 variant of the MOLLI sequence has been shown to be heart rate dependent, and while the 5s(3s)3s is an improvement, some remaining heart rate dependence is to be expected.^21^ The error introduced at high heart rates is due to incomplete relaxation and residual longitudinal magnetization at the time of the next inversion, leading to a lower measured T_1_ value. The amount of residual longitudinal magnetization is not only increased at high heart rates but also at higher T_1_ values. Notably, the native T_1_ values for pericardial fluid in the current study did not vary with heart rate as shown in *Figure 2*. As patients with haemodynamically significant pericardial effusion frequently have elevated heart rates, the impact of heart rate on T_1_ values in pericardial fluid should be addressed in future studies with larger populations.

In one study, 0.6% of CMR exams were found to have incidental findings of clinically significant pericardial effusion.^22^ A non-invasive alternative to diagnostic pericardiocentesis would have clinical significance as all invasive procedures involve some degree of risk. International guidelines recommend pericardiocentesis with biochemical testing upon suspicion of bacterial or neoplastic pericardial effusion.^23^ A non-invasive method for characterizing pericardial fluid as transudate, and hence not of bacterial or neoplastic aetiology, could then be used to avoid unnecessary diagnostic pericardiocentesis. If, on the other hand, a pericardial effusion is characterized as an exudate, CMR imaging provides detailed anatomical information highly relevant to the pericardiocentesis procedure itself.

### Limitations

A limitation of this study is that it does not include any data on pathology or demonstration of the usefulness of this approach to characterize the nature of the effusion, and future such studies are justified.

The study population is relatively small with a large range of ages. However, there was no association between pericardial fluid T_1_ values and age or sex, and the results from the 26 subjects reported in the results are an adequate sample size to assume a normal distribution and determine a normal range.

A potential limitation of the current study is the size of ROI required to measure the small volumes of normal pericardial fluid present in healthy volunteers. The matrix size, field of view, and slice thickness used in this study are equal to those in routine clinical use. The median ROI size of 18 mm^2^ in this study translates to 8 in-plane voxels, or image pixels, per ROI whereas international CMR consensus guidelines recommend avoiding ROI sizes below 20 image pixels for T_1_ mapping of the myocardium.^24^ Even though some partial volume is inevitable, we attempted to overcome this limitation by employing a minimum and maximum ROI size criteria, a within-ROI variance criteria, and a between-ROI variance criteria with separately acquired and perpendicularly oriented measurements. There are currently no studies or guidelines available to inform the choice, or specific values, of the above criteria for measurements of T_1_ in pericardial fluid. They were chosen for purposes of consistency based on clinical judgement.

The T_1_ values of the pericardial fluid of healthy volunteers measured in this study are considerably higher than the proposed validated range for measuring native T_1_ (below 1200 ms).^21^ An underestimation of T_1_ values is thus to be expected, and the normal values presented in this study should not be generalized beyond the specific 5s(3s)3s variant of the MOLLI sequence. To our knowledge, there are no simulation, phantom, or volunteer studies that investigate the accuracy and precision of cardiac T_1_ mapping sequences for the higher T_1_ values of serous fluids.

Although potentially of interest, this study did not include T_2_ mapping characteristics of the pericardial fluid of healthy volunteers.

## Conclusion

T_1_ can be reliably measured at 1.5 T in a normal volume of pericardial fluid in healthy volunteers, and normal reference values are presented. The use of native T_1_ mapping to characterize pericardial fluid is currently in its infancy, and further research is required to determine the value of this approach. The precision and accuracy of specific cardiac T_1_ mapping sequences for measurement of the higher T_1_ values of serous fluids is an open question.

## Data Availability

The data sets analysed in the current study are available from the corresponding author upon reasonable request.

## Lead author biography

Simon Thalén is a resident physician in clinical physiology and a PhD candidate at Karolinska University Hospital and Karolinska Institutet in Stockholm, Sweden. He has a past in mathematics and philosophy prior to attending medical school. His thesis involves evaluation of constrictive physiology in pericardial disease using cardiovascular magnetic resonance (CMR). His areas of expertise include cardiology, CMR, and software development.
